# Chemical Data and Relationships for a Scoring Algorithm of Extra Virgin Olive Oil’s Nutritional Value

**DOI:** 10.3390/molecules29020525

**Published:** 2024-01-21

**Authors:** Lorenzo Cecchi, Filippo Conticelli, Bruno Zanoni, Carlotta Breschi, Maria Bellumori, Nadia Mulinacci

**Affiliations:** 1 Department of Agriculture, Food, Environment and Forestry (DAGRI), University of Florence, Piazzale Delle Cascine 16, 50144 Florence, Italy; bruno.zanoni@unifi.it (B.Z.); carlotta.breschi@unifi.it (C.B.); 2 Placet srls, Via Donizetti 21/1, Empoli, 50053 Florence, Italy; fconticelli@icloud.com; 3 Department of Neuroscience, Psychology, Drug and Child Health, Pharmaceutical and Nutraceutical Section, University of Florence, Via Ugo Schiff 6, Sesto Fiorentino, 50019 Florence, Italy; maria.bellumori@unifi.it (M.B.); nadia.mulinacci@unifi.it (N.M.)

**Keywords:** phenolic compounds, tocopherols, fatty acids composition, nutritional quality, EFSA health claim, olive oil polyphenols, nutraceutical, oxidative stability, Nutri-Score, antioxidants

## Abstract

Extra virgin olive oil (EVOO) is a valuable product and is highly appreciated by consumers for its great nutritional value. However, to date, there has been a lack of uniform systems capable of ranking the nutritional value of EVOO based on its chemical composition in terms of macro- and micronutrients (including phenolic compounds and tocopherols). The aim of this study was to propose a scoring algorithm to rank the nutritional value of EVOO samples, considering their chemical composition in macro- and micronutrients and their sensitivity to oxidation phenomena. Data from more than 1000 EVOO samples were used to assess the variability of the data, considering the selected negative parameters (free acidity, peroxide value, spectrophotometric indices) and positive components (composition in tocopherols via HPLC-DAD, phenolic compounds via HPLC-DAD, and fatty acids via GC-MS) so as to ensure the universal validity of the scoring algorithm. The dataset included samples from the main producing countries worldwide, in addition to Australia, across several production years; data were selected to represent different production realities. A mathematical model was set up for each chemical component, resulting in six variable values. By combining these values with a dimensionless constant value, the algorithm for computing the nutritional value score (NVS) was defined. It allows the nutritional value of an oil to be ranked on a scale of 0 to 100 based on its chemical composition. The algorithm was then successfully tested using chemical data from about 300 EVOO samples obtained from laboratories from different Italian regions. The proposed NVS is a simple and objective tool for scoring the nutritional value of an EVOO, easy to understand for both producers and consumers.

## 1. Introduction

There are a great variety of edible oils, originating from different botanical sources (e.g., seeds, nuts, fruits) and with very different nutritional values [1,2]. Among them, olive oil, particularly virgin olive oil (VOO), is widely recognized as the oil with the best nutritional value, mainly due to the combination of its fatty acid composition and of the presence of antioxidants [1,3,4,5] such as hydrophilic phenolic compounds (e.g., secoiridoid, lignans, flavonoids, phenolic acids and phenolic alcohols; hereinafter, phenolic compounds), and lipophilic phenolic compounds such as tocopherols (i.e., vitamin E). Among VOOs, which are “the oils obtained from the fruit of the olive tree (*Olea europaea* L.) solely by mechanical or other physical means under conditions […] that do not lead to alterations in the oil […]” [6,7], extra virgin olive oil (EVOO) is the premium commercial category. Oils belonging to the EVOO category must conform to several chemical and sensory legal limits, including the absence of sensory defects, to confirm their authenticity [8].

To date, product certification schemes based on EVOO nutritional standards are lacking, despite EVOO being widely recognized in the scientific community as a nutraceutical food. A nutraceutical food is usually defined as a natural product able to provide extra health benefits in addition to those provided by the well-known macro- and micronutrients, including the prevention of a disease by reducing disease risk factors, and being defined as between a food and a drug [9]. Generally speaking, any food containing antioxidant molecules and with a low oxidative status has a nutraceutical effect on human health. Some recommendations based on the oil composition of edible oils are available from international organizations [10,11,12], highlighting the molecules linked to EVOO’s nutraceutical value. For example, the European Food Safety Authority (EFSA) has approved a list of health claims pertaining to EVOO [13,14], including two health claims related to substances which are present in EVOO as well as elsewhere (i.e., tocopherols and unsaturated fatty acids) and a specific health claim for substances present only in EVOO (i.e., secoiridoid phenolic compounds): (i) it contains olive oil polyphenols, which contribute to the protection of blood lipids from oxidative stress; (ii) it is rich in vitamin E, which contributes to the protection of cells from oxidative stress; (iii) it is rich in unsaturated fatty acids, mainly oleic acid; replacing saturated fats with unsaturated fats in the diet contributes to the maintenance of normal blood cholesterol levels. However, these health claims are still scarcely used by EVOO producers and bottlers, since they are presumed not to be clearly understandable for the consumer [3,15].

Front-of-pack labels (FoPLs) are considered a policy tool to drive consumers toward healthy food choices. Nutri-Score and NutrInform represent the latest examples of nutritional labels for food and beverages in the European Union [16]. However, the widespread application of FoPLs for dietary oils is still controversial, since the algorithms only consider the macronutritional composition, such as the presence of saturated fatty acids. A specific ranking system that gives an overall nutritional score of EVOO may be useful to develop certification schemes which add value to the product and communicate easy-to-understand information for consumers. Recently, some authors proposed models for ranking the nutritional values of different vegetable oils and showed that VOO is at the top [1,17]. However, none of the studies included the typical phenolic compounds of EVOO in their scoring models.

Therefore, in this study, an original scoring algorithm was developed to rank the nutritional value of EVOOs based on a very large dataset. The algorithm was set up to give importance both to all the main nutritional chemical compounds and to the sensitivity of the oil to oxidation phenomena during shelf life.

## 2. Results and Discussion

The following multistep procedure was followed to develop the algorithm for computation of the EVOO nutritional value score (NVS):

1. Selection of the chemical components to be included in the algorithm.
2. Evaluation of the data variability for each component across the virgin olive oil samples.
3. Set up of the NVS algorithm through a mathematical model for each chemical component.
4. Testing of the NVS algorithm.

### 2.1. Selection of the Chemical Components

The first requirement for a virgin olive oil sample to be included in the NVS computation is that it must comply with the European EVOO legal limits [8]. Therefore, the limits of the chemical characteristics must be met, both to ensure that good manufacturing practices are applied (e.g., free acidity, peroxide value) and to avoid commercial fraud (e.g., fatty acid composition, sterol content and composition). The limits of the sensory characteristics must also be observed (i.e., the median of the fruit must be greater than zero and the median of the defect must be zero).

For the development of the algorithm, chemical components that can positively or negatively influence the nutritional quality of EVOO were selected. The selected components that negatively affect the nutritional quality of the EVOO sample (i.e., the NCs) are free acidity, peroxide value, and UV spectrophotometric indices, while those positively influencing the nutritional quality (i.e., the PCs) are fatty acid composition, phenolic compounds, and tocopherols. The rationale behind this choice is described in more detail in Section 3.1.

### 2.2. Evaluation of the Data Variability

In order to create the databases for setting the algorithm, the evaluation of the data variability of the above chemical components across more than 1000 virgin olive oil samples was carried out. Data were from different olive oil production years characterized by different conditions including geographic area; in the following discussion, they are reported as percentages of samples within specific ranges of the considered parameter to depict the variability of the data so as to ensure universal validity of the scoring algorithm developed.

#### 2.2.1. Free Acidity (NC1), Peroxide Value (NC2), and UV Spectrophotometric Indices (NC3)

**Figure 1:**
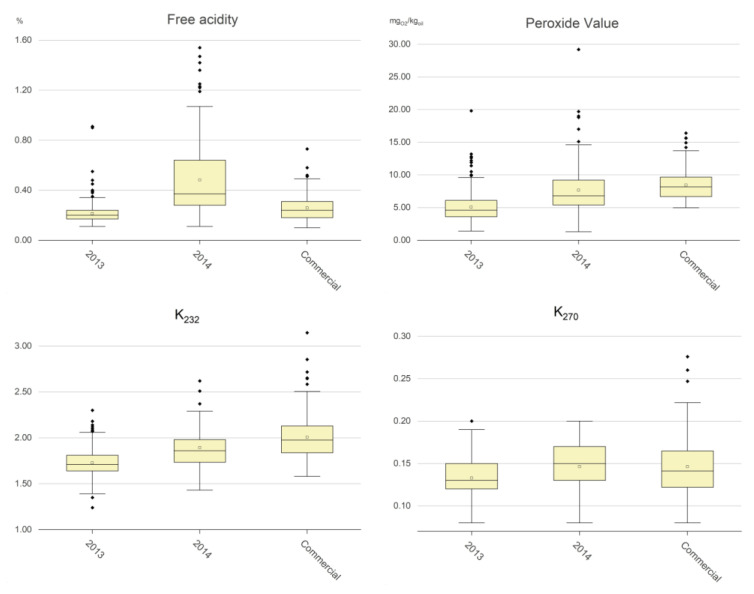
Boxplots of the amounts of negative chemical components in the EVOO samples. The empty squares are the mean values and the full black rhombi are outlier values.

Table 1 and Figure 1 show the comparison of the values of the negative components between the virgin olive oil samples, expressed as classes of values and represented as boxplots, respectively.

Free acidity (% oleic acid): The legal limit for the EVOO commercial category is 0.80% [8]. It is well known that when sound olives are correctly treated during virgin olive oil extraction such a limit is reached only in a very few cases, as confirmed by the experimental data: only 2 out of 502 samples from olive oil production years free from olive fly attacks (i.e., the 2013 samples and the commercial samples) had free acidity values greater than 0.8%. Even in a production year with a very strong fly attack (i.e., 2014), the number of samples with a free acidity higher than 0.8% was not very high (i.e., 31 out of 220 samples). The boxplots in Figure 1 highlight that free acidity was strongly affected by olive fruit fly attack, which led to a mean value of 0.50% for the 2014 samples, and to quite a high number of samples with values greater than 0.50%. The mean value was much lower in the 2013 samples and in the commercial samples (i.e., 0.21% and 0.26%, respectively). Summarizing the above experimental data, we can state the following:

- The free acidity was lower than 0.25% for approx. 80% of the olive oil samples produced by small local producers in an olive oil production years free from olive fly attack or other adverse conditions (i.e., the 2013 samples); the percentage of samples with free acidity lower than this value dropped to 53.4% for the commercial samples, and to 17.7% for the samples produced in an olive oil production years with a strong olive fly attack (i.e., the 2014 samples).
- The free acidity was greater than 0.50% for 35.9% of the 2014 samples, for 2.8% of the commercial samples and for a negligible number of the 2013 samples.

Peroxide value (meq_O2_/kg_oil_): The legal limit for the EVOO commercial category is 20 meq_O2_/kg_oil_ [8]. This value can increase in the presence of oxygen and, in particular, when a sample is exposed to light, but it can decrease due to the breakage of the hydroperoxides to form shorter volatile molecules linked to the rancid defect [18,19,20,21]. The experimental data (Table 1) highlighted that neither the 2013 samples nor the commercial samples exceeded the limit for the EVOO category; only 0.5% of the 2014 samples did so. The boxplots in Figure 1 point out that the peroxide values were lowest for the 2013 samples, with a mean value of 5.1 meq_O2_/kg_oil_; there were little difference between the 2014 samples and the commercial samples (i.e., 7.7 and 8.4 meq_O2_/kg_oil_, respectively). Summarizing the above experimental data, we can state the following:

- The peroxide value was lower than 3 meq_O2_/kg_oil_ for only 7.1% of the 2013 samples, for 2.9% of the 2014 samples, and for none of the commercial samples.
- The peroxide value was higher than 10 meq_O2_/kg_oil_ for only 2.6% of the 2013 samples, while the percentage was greater for the 2014 samples and commercial samples (i.e., 20.5 and 19.7%, respectively).
- The peroxide value was greater than 15 meq_O2_/kg_oil_ for a negligible number of the 2013 samples, and for 3.4 and 2.0% of the 2014 samples and commercial samples, respectively.

UV spectrophotometric indices: The legal limits of the K_232_ and K_270_ for the EVOO commercial category [8] are 2.50 and 0.22, respectively. The values indicate oil secondary oxidation level and any formation of conjugates trienes due to refining processes. They can increase over storage time but, differently from the peroxide value, they cannot decrease. The experimental data (Table 1) showed that none of the 2013 samples overcame the limits for the EVOO category, that 2.6% of the 2014 samples overcame both the K_232_ and K_270_ limits, and that 5.4 and 2.7% of the commercial samples overcame the K_232_ and K_270_ limits, respectively. The boxplots in Figure 1 point out that the values of K_232_ were the lowest for the 2013 samples, with a mean value of 1.73, and that the greatest values were for the 2014 samples (1.89) and for the commercial samples (2.01). The values of K_270_ were still the lowest for the 2013 samples (mean, 0.13), but with smaller differences in comparison to the 2014 samples and the commercial samples (0.16 and 0.15, respectively). Summarizing the above experimental data, we can state the following:

- The K_232_ values were lower than 1.50 for 5.5% of the 2013 samples, for 1.3% of the 2014 samples, and for none of the commercial samples.
- The K_232_ values were greater than 1.85 for 17.5% of the 2013 samples, while the percentage was the greatest for the 2014 samples and the commercial samples (55.3 and 72.1%, respectively).
- The K_232_ values were greater than 2.15 for only 0.9% of the 2013 samples, and for 15.8 and 23.1% of the 2014 samples and the commercial samples, respectively.
- The K_270_ values were lower than 0.10 for 3.4% of the 2013 samples, for 3.9% of the 2014 samples, and for 3.4% of the commercial samples.
- The K_270_ values were greater than 0.17 for 10.3% of the 2013 samples, and for 27.6 and 21.1% of the 2014 samples and the commercial oils, respectively.
- The K_270_ values were greater than 0.20 for only 0.9% of the 2013 samples, and for 4.1 and 6.1% of the 2014 samples and the commercial oils, respectively.

All above experimental data relating to the selected negative chemical components indicated that free acidity was strongly and negatively affected by adverse agronomic events such as olive fly attacks, whereas the peroxide value and the spectrophotometric indices were more sensitive to storage conditions of the commercial samples.

#### 2.2.2. Fatty Acid Composition (PC1), Phenolic Compounds (PC2), and Tocopherols (PC3)

**Figure 2:**
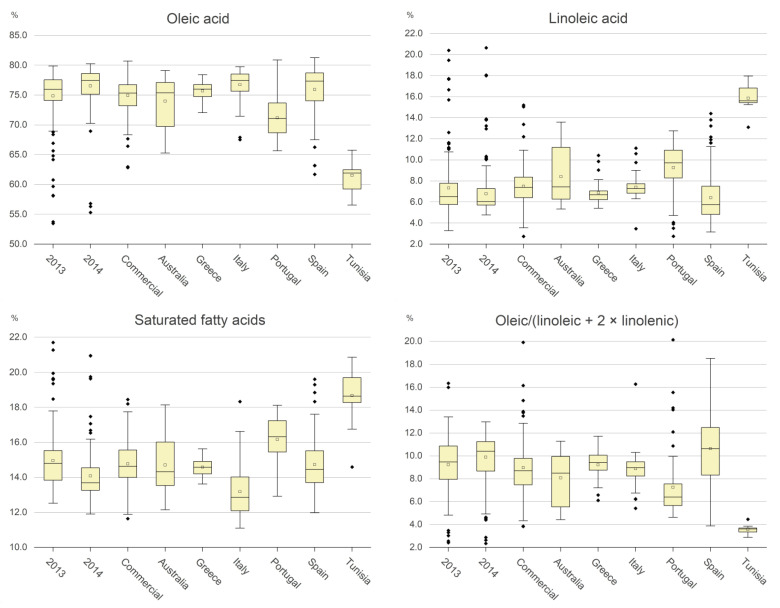
Boxplot of the fatty acids included in the algorithm. The empty squares are the mean value; the full black rhombi are outlier values.

**Figure 3:**
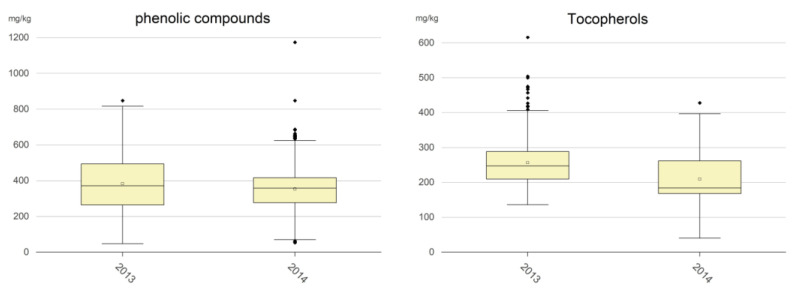
Boxplot of hydrophilic phenolic compounds and tocopherols. The empty squares are the mean values; the full black rhombi are the outlier values.

The legal limits of each of the fatty acids present in the olive oil for the EVOO commercial category were recently modified [8], as follows: miristic acid < 0.03%; palmitic acid: 7.00–20.00%; palmitoleic acid: 0.30–3.50%; margaric acid < 0.4%; margaroleic acid: < 0.6%; stearic acid: 0.50–5.00%; oleic acid (OA): 55.00–85.00%; linoleic acid (LA): 2.50–21.00%; linolenic acid (LnA): <1.00%; arachidic acid: <0.60%; eicosenoic acid: <0.50%; behenic acid: <0.20%; lignoceric acid: <0.20%; Trans C18′ < 0.05%; Trans C18″ + C18‴ < 0.05%. Table 2 and Table 3 and Figure 2 and Figure 3 show a comparison of the amounts of positive components between virgin olive oil samples, expressed as classes of values and represented as boxplots, respectively.

Fatty acid composition (%)

The bulk mass of EVOO is almost entirely (≈98%) constituted by triglycerides, which are in turn constituted by three molecules of fatty acids linked to glycerol via esterification of the alcoholic and carboxylic acid functions.

Some fatty acids are present in very low percentages and can be considered negligible from a nutritional standpoint; they were not considered in the computation of the algorithm and are miristic, palmitoleic, margaric, margaroleic, arachidic, eicosenoic, behenic, lignoceric, and the trans forms of the C18 unsaturated acids. The fatty acids and their combination that were instead included in the algorithm were as follows:

1. Oleic acid (%): this monounsaturated acid demonstrated the most benefits for human health, in combination with the minor compounds of the EVOO.
2. Saturated fatty acids (%):saturated fatty acids are known for their negative contribution to human health. Therefore, increasing amounts of these molecules will be included in the algorithm as a way to reduce the positive contribution of the fatty acid composition.
3. The ratio [OA/(LA + 2 × LnA)]: polyunsaturated fatty acids (PUFAs) contribute to human health with two opposing effects. On the one hand, they belong to the families of ω3 (linolenic acid—LnA) and ω6 (linoleic acid—LA), which are essential fatty acids with a positive contribution to the nutritional quality of oil. On the other hand, they are much more susceptible to oxidation than saturated and monounsaturated fatty acids [19,22] thus providing a negative contribution. It should also be considered that, in EVOO, they are usually present in quite low percentages, and that linoleic acid largely exists in linolenic acid, the latter being the PUFA with the most positive properties. On this basis, in addition to the fact that small amounts of EVOO should be taken daily, EVOO cannot be considered a source of ω3, and the contribution of PUFA to the algorithm was only considered (in combination with oleic acid) in terms of oxidative stability by the parameter given by the formula: OA/(LA + 2 × LnA), where the contribution of LnA was multiplied by two to take into account its double rate of oxidation with respect to LA [22]. The higher the value of this parameter, the higher the oil’s stability against oxidation.

The variability of both the above three components and the linoleic acid for the 2013, 2014 and commercial samples is shown in the boxplots in Figure 2, whereas Table 2 shows the percentage of oils within specific ranges of the components. Overall, a very negligible number of oils showed values out of the limits for the EVOO category for all the fatty acids. The oleic acid was more affected by geographic origin than by adverse effects such as olive fly attacks. It exhibited highest values in the Italian, Greek, and Spanish samples, followed by the Australian and Portuguese ones, while the Tunisian oil exhibited much lower percentages of this acid. A fairly opposite behavior was observed for linoleic acid. The saturated fatty acids exhibited a high variability among the classes, with the highest and the lowest values being in Tunisian and Italian oils, respectively. The ratio OA/(LA + 2 × LnA) showed the lowest values for the Tunisian samples, while the Spanish oils were those with the highest mean values, but they also showed a high variability. Summarizing the above experimental data, we can state the following:

- Values of saturated fatty acids lower than 8 or higher than 25 are not permitted in that they imply that at least one of the two acids is outside of the EVOO legal limits.
- The value of this parameter is in the range 11–20% for almost all the oils in the dataset, with the exception of the Tunisian oils, 14% of which had contents greater than 20%.
- Values of oleic acid outside of the range 55–85% are not permitted, as they are out of the EVOO legal limits.
- For samples from the 2013 and 2014 production years, commercial samples and Italian, Spanish, and Greek samples, oleic acid is almost always greater than 71%. In the case of the Tunisian samples, no oils had oleic acid in concentration greater than 71%.
- Values of the ratio (OA/(LA + 2 × LnA)) lower than 2.4 or greater than 34.0 are not permitted, as they imply that at least one of the three acids is out of the EVOO legal limits.
- For the other values, the greater the ratio, the greater the oxidative stability of the oil, and therefore the lower the negative contribute of the value to the NSV.

Phenolic compounds (mg/kg): Despite phenolic compounds’ strongly contribution to the nutritional properties of EVOO, no legal limits for the different commercial categories have been established by EU regulation, and an oil can be classified as EVOO even if its content in these compounds is very low. Figure 3 shows that only slight differences were pointed out in terms of mean values of the phenolic content among the 2013 samples and 2014 samples. Table 3 shows that only a negligible percentage of samples had phenolic contents below 50 mg/kg and that approx. 17–20% of oils had phenolic contents below 250 mg/kg, that is the limit established by the EFSA for allowing an EVOO to use the health claim for virgin olive oil phenolic compounds [14,23]. A great number of samples had phenolic contents in the range 250–500 (i.e., 55.8% of the 2013 samples and 72.7% of the 2014 samples), whereas the content was in the range 500–800 mg/kg for 22.7% and 9.4% of the 2013 samples and 2014 samples, respectively. Finally, only 0.7% of the 2013 samples and 0.4% of the 2014 samples showed values greater than 800 mg/kg.

Tocopherols (mg/kg): Also in this case, no legal limits for the different commercial categories have been established by EU regulation. Figure 3 shows that the tocopherol content was more sensitive to adverse situations such as olive fly attacks than the phenolic compounds content. Indeed, the mean value for the 2014 samples was 209 mg/kg, quite significantly lower than the 257 mg/kg mean value of the 2013 samples. Furthermore, 81.6% of the 2013 samples had a tocopherol content greater than 200 mg/kg, whereas this was only the case for 42.6% of the 2014 samples (Table 3). Finally, a low percentage of samples showed a content greater than 400 mg/kg and a negligible percentage greater than 500 mg/kg.

### 2.3. Setting Up of the NVS Algorithm

**Figure 4:**
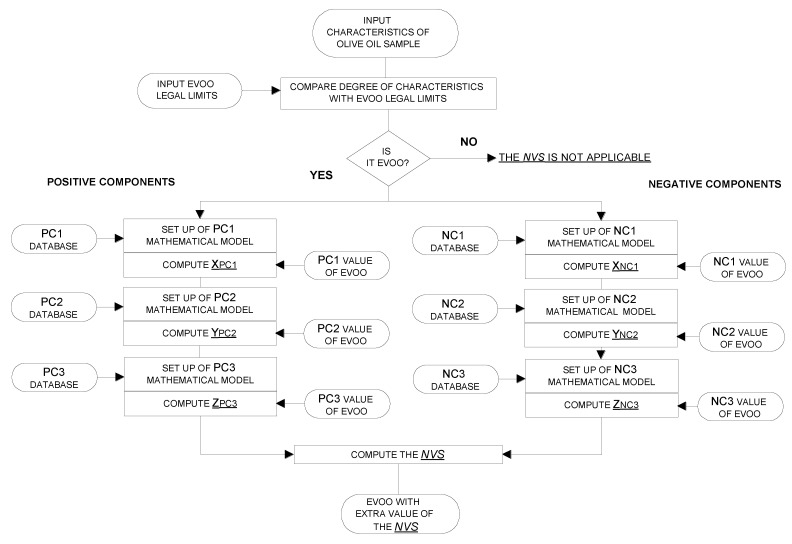
Flow chart of the algorithm.

Figure 4 shows the flow chart of the algorithm for the EVOO NVS. The algorithm starts with the comparison of the chemical and sensory characteristics degree of the sample with the legal limits (see paragraph 2.1); if only one of these characteristics is not-conforming, the sample is excluded from the NVS. Then, the algorithm computes the dimensionless values of the positive and negative chemical components of Equation (1), which is described in Section 3.1. The above dimensionless values resulted from mathematical models, which were based on the components databases previously described in terms of percentages of samples within specific ranges of the considered parameters. The mathematical models were linear and non-linear equations designed on the basis on the percentage of samples with specific range of values as reported in Table 1, Table 2 and Table 3 and having as boundary conditions either the legal limits or specific values selected on the basis of the data in Table 1, Table 2 and Table 3. Via this method, for example, in the case of free acidity, the maximum negative contribution was already reached at values well below the legal limit, as this value (i.e., 0.8%) is only rarely reached when strong olive fly attacks occur (see in Table 1) and the non-linear equation was designed to give an increasingly negative score to the NVS following the values of percentage of samples with specific ranges (Table 1).

#### 2.3.1. Mathematical Models for the Positive Components

Table 4 shows an overview of the mathematical models applied to the positive components of NVS.

The dimensionless value (X_PC1_) of the fatty acid composition (PC1) of EVOO samples was computed using the sum of the values for the oleic acid content (X_OA_), for the saturated fatty acids content (X_SFA_), and for the ratio OA/(LA + 2 × LnA) (X_RATIO_). The contribution of the oleic acid content to the NVS is the highest, as oleic acid content differentiates EVOO from almost all other edible oils (the only exception is peanut oil). The contribution to the X_OA_ value was maximum and minimum for values close to the upper and lower legal limits of oleic acid for the EVOO category, respectively; within the limits for the EVOO category, the X_OA_ value is computed via a sigmoidal equation. The contribution of the saturated fatty acids to the NVS is to reduce the positive contribution of the fatty acid composition. The negative contribution to the X_SFA_ value is maximum and minimum for values close to the upper and lower legal limits of palmitic + stearic acids for the EVOO category, respectively; within the limits for the EVOO category, the X_SFA_ value is computed via a sigmoidal equation. The contribution of the ratio OA/(LA + 2 × LnA) to the NVS is to decrease the positive contribution of the fatty acid composition while decreasing the ratio. The decreasing contribution to the X_RATIO_ value is maximum and minimum for, respectively, the minimum and maximum values of the ratio allowed by the legal limits for the EVOO category for the three fatty acids involved in the formula (i.e., oleic acid, linoleic acid, and linolenic acid); between the above values, the X_RATIO_ value is computed via a sigmoidal equation.

The dimensionless value (Y_PC2_) of the phenolic compounds (PC2) of EVOO samples is zero at a phenolic content equal to 0 mg/kg; it is computed via two sigmoidal equations which have different proportional constants for values of phenolic contents smaller or greater than 250 mg/kg, to give a small score for values below 150 mg/kg and an extra score to samples with phenolic content greater than the limit established by the EFSA for the EVOO phenolic compounds health claim [23].

The dimensionless value (Z_PC3_) of the tocopherols (PC3) of EVOO samples is 0 at tocopherol content of 0 mg/kg; then, the Z_PC3_ value is computed via two linear equations in order to have the fastest rate of increase in Z_PC3_ with increasing the tocopherol content.

#### 2.3.2. Mathematical Models for the Negative Components

Table 5 shows an overview of the mathematical models applied to the negative components of NVS.

The dimensionless value (X_NC1_) of free acidity (NC1) of EVOO samples is maximum at free acidity ≥ 0.50% and it was zero at free acidity ≤ 0.10%. Between the above values, the X_NC1_ value is computed via a sigmoidal equation.

The dimensionless value (Y_NC2_) of the peroxide value (NC2) of EVOO samples is maximum at values ≥ 18 meq_O2_/kg and is zero at values ≤ 3 meq_O2_/kg. Between the above values, the Y_NC2_ value is computed via three linear equations in order to achieve the fastest rate of increase in Y_NC2_ with increasing peroxide values.

The dimensionless value (Z_NC3_) of the UV spectrophotometric indices (NC3) of EVOO samples is computed by the sum of values for the K_232_ (Z_K232_) and K_270_ (Z_K270_). The contribution to the Z_K232_ value is maximum at K_232_ ≥ 2.45 and was zero at K_232_ ≤ 1.50; between the above values, the Z_K232_ value is computed via a sigmoidal equation. The contribution to the Z_K270_ value is maximum at K_270_ ≥ 0.20 and is zero at K_270_ ≤ 0.10; between the above values, the Z_K270_ value is computed via a sigmoidal equation.

### 2.4. Testing of the NVS Algorithm

The NVS algorithm was thought to give the NVS value of 0 for EVOOs with minimum nutritional value and the NVS value of 100 for EVOOs with the best combination of the beneficial effects on human health and oxidation stability. The balance of the dimensionless values for positive and negative components leads to good (when X_PC1_ + Y_PC2_ + Z_PC3_ prevail on the negative ones) or bad values (when X_NC1_ + Y_NC2_ + Z_NC3_ prevail on the positive ones) of the NVS.

**Figure 5:**
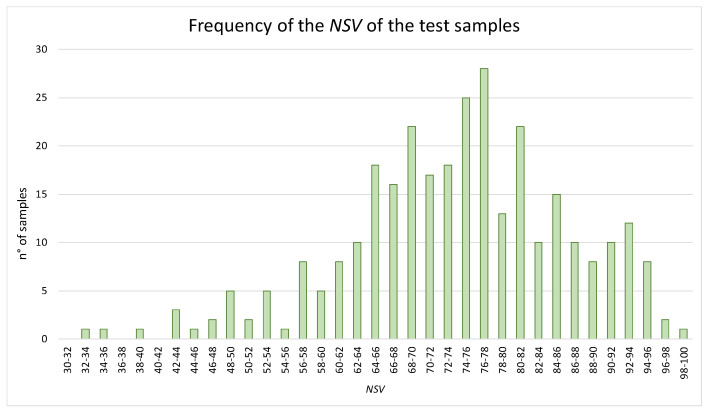
Bar chart of the frequency of the NSV in different ranges for the samples of the test set.

For the purposes of testing the NVS algorithm, values of the positive and negative components of 308 EVOO samples were collected from several Italian analytical laboratories; Figure 5 shows the relevant NVS distribution of the above samples.

Most of the samples fell in the NVS range of 60–80, only a low number of samples reached the excellency (i.e., NSV ≥ 96), and some samples reached quite low values (up to NVS = 32). The distribution of the NVS was considered satisfactory as it was similar to a Gaussian curve.

## 3. Materials and Methods

### 3.1. The Theory behind the Algorithm

The scoring algorithm of EVOO nutritional value was based on the following assumptions:

- Belonging to the premium commercial category of EVOO is mandatory. If only one of the European legal chemical and sensory characteristics does not conform with the relevant legal limit [8], the olive oil sample is excluded from the NVS. The NVS is an extra value to the EVOO commercial category.
- The NVS is determined using the model in Equation (1). The model combines a dimensionless constant value (K) and six variable values, of which three dimensionless values (X_PC1_, Y_PC2,_ Z_PC3_*)* have positive weight and three dimensionless values (X_NC1_, Y_NC2,_ Z_NC3_) have negative weight on the NVS:

(1) $$\mathrm{NVS}=K+\left(X_{\mathrm{PC}1}+Y_{\mathrm{PC}2}+Z_{\mathrm{PC}3}\right)-\left(X_{\mathrm{NC}1}+Y_{\mathrm{NC}2}+Z_{\mathrm{NC}3}\right)$$

The X_PC1_, Y_PC2_, and Z_PC3_ values functions of the content of three groups of compounds (i.e., the PC1, PC2, and PC3 positive components), which have nutritional properties. Instead, the X_NC1_, Y_NC2_, and Z_NC3_ values are a function of the amount of three parameters (i.e., the NC1, NC2, and NC3 negative components) which explain EVOO’s sensitivity to oxidation phenomena during shelf life. Therefore, as the NVS increases, the combination of the beneficial effects on human health and of the oxidation stability of EVOO increase. The above positive and negative values are described below. The NVS is scored on a scale of 0–100 and the K value was included to prevent NVS values below zero.

#### 3.1.1. The Positive Components

The following positive components were selected as those most linked to the nutritional properties of the EVOO and that most differentiate EVOO from the other vegetable oils: (i) the fatty acid composition (PC1); (ii) the secoiridoid phenolic compounds (PC2); (iii) the tocopherols (PC3).

The fatty acid composition of olive oil is unique among edible oils such that the high content of oleic acid allows for the use of the health claim “the olive oil is rich in unsaturated fatty acids”, according to EU regulations [13,14,24]. The high oleic acid content value has been also found to indicate oils less susceptible to lipid oxidation than oils rich in polyunsaturated fatty acids (PUFA) [19,22,25]. Therefore, the percentage content of oleic acid, the percentage content of saturated fatty acid, and the ratio of oleic acid to PUFA content were included in the scoring algorithm via the parameter *PC1*. The secoiridoid phenolic compounds are even more unique in VOO, also because VOO is extracted by olive fruits and not by seeds and their intake can be only achieved by consuming EVOO [26,27,28]. As antioxidant compounds, the secoiridoids also have an inhibiting effect on lipid oxidation. Tocopherols were included because their level of occurrence in EVOO allows the use of the claim “rich in vitamin E”, according to EU regulations [13,14,29]. Since tocopherols are also present in many other kinds of edible oils [4], and their amount is sometimes higher than that in EVOO (e.g., sunflower oil, albeit refined, can contain a concentration of tocopherols 2.5–3.5 greater), the significance of the fatty acid composition and the secoiridoid phenolic compounds on the NVS was chosen to be higher than that of tocopherols.

Phytosterols were not included in the NVS. Their total content in EVOO is below 2000 mg/kg, meaning that approx. 400 g/die of EVOO must be consumed to reach the 0.8 g daily intake recommended in the literature to obtain beneficial effects [4,30,31]. Therefore, the contribution of sterol fraction to the EVOO nutritional value was considered negligible, with respect to secoiridoid phenols and tocopherols.

#### 3.1.2. The Negative Components

The following negative components were selected as being those most negatively linked to the oxidative stability of EVOO: (i) the free acidity (NC1); (ii) the peroxide value (NC2); (iii) the spectrophotometric indices K_232_ and K_270_ (NC3). Free fatty acids are more susceptible to oxidation than their corresponding esterified fatty acids; therefore, the free acidity value is related to the susceptibility of the oil to oxidation [22,32,33]. Peroxides are the main initial compounds formed by autoxidation, a negative phenomenon that also causes the shift of double bonds of polyunsaturated fatty acids such as linoleic and linolenic acids. During peroxides’ formation, the chemical structure of fatty acids rearranges to form conjugated double bonds that determine absorption at 232 nm and 268/270 nm. Therefore, the peroxide value and the spectrophotometric indices are related to the oxidative status of the oil [22,33]. The significance of all the above negative components on the NVS was chosen as equal.

Concerning ΔK, that is the third UV spectrophotometric index included among the legal quality parameters for assessing the commercial category of VOO [8], it is used to verify whether the oil belongs to the EVOO category or not, and if the oil is consequently suitable for the computation of the NVS, but it does not contribute to the final score.

### 3.2. Data for the Algorithm Constitutive Equations

Determination of the value in Equation (1) was based on mathematical models:

(2) $$X,Y,Z_{\mathrm{PC}1,\;\mathrm{PC}2,\;\mathrm{PC}3}=f\left(\mathrm{PC}1,\;\mathrm{PC}2,\;\mathrm{PC}3\right)$$

(3) $$X,Y,Z_{\mathrm{NC}1,\;\mathrm{NC}2,\;\mathrm{NC}3}=f\left(\mathrm{NC}1,\;\mathrm{NC}2,\;\mathrm{NC}3\right)$$

which were set up by means of a database consisting of many experimental data from olive oil samples for each positive and negative component. The olive oil samples were selected in order to represent different production realities, as follows:

1. **The 2013 samples**: Samples from small Italian producers during an olive oil production year (i.e., the 2013) free from olive fly attack or other adverse conditions, such as pathogens attack and adverse meteorological conditions; they represented the situation in which the oil characteristics were not subject to different changes than those naturally occurring in olive oil samples.
2. **The 2014 samples**: Samples from small Italian producers during an olive oil production year (i.e., the 2014) characterized by a strong attack of olive fruit flies [34]; they represented the situation in which the oil characteristics were subject to negative changes due to the poor sanitary conditions of olive fruits.
3. **The commercial samples**: Commercial samples from different olive oil production years; they represented the situation in which the oil characteristics were potentially subject to inappropriate storage conditions (i.e., storage conditions different from those recently recommended by the International Olive Council [35]). Experimental data from the commercial samples were not available for tocopherol or phenolic compounds’ contents. In the case of the fatty acid compositions, additional experimental data were also included from commercial samples with different geographic origins.

In summary, for each parameter, the database consisting of the following experimental data:

- Free acidity. Experimental data were collected from a total of 722 samples: 356 samples from the 2013 production year, 220 samples from the 2014 production year, and 146 commercial samples.
- Peroxide value. Experimental data were collected from a total of 711 samples: 354 samples from the 2013 production year, 210 samples from the 2014 production year, and 147 commercial samples.
- Spectrophotometric indices. Experimental data were collected from a total of 458 samples: 235 samples from the 2013 production year, 76 samples from the 2014 production year, and 147 commercial samples.
- Phenolic compounds. Experimental data were collected from a total of 1169 samples: 564 samples from the 2013 production year, and 605 samples from the 2014 production year.
- Tocopherols. Experimental data were collected from a total of 861 samples: 464 samples from the 2013 production year, and 397 samples from the 2014 production year.
- Fatty acid composition. data were gathered from a total of 840 samples: 173 samples from the 2013 production year, 179 samples from the 2014 production year, 146 commercial samples, and 342 commercial samples from different declared geographic origin (i.e., 149 Spanish, 64 Italian, 62 Portuguese, 36 Greek, 16 Australian, 14 Tunisian, and 1 Peruvian).

### 3.3. Data for the Algorithm Testing

A further set of data from 308 EVOO samples, collected from several Italian laboratories, was used for testing the algorithm. For these samples, the data relating to all the parameters included in the algorithm (i.e., free acidity, peroxide value, spectrophotometric indices, fatty acid composition, tocopherols, phenolic compounds) were available.

### 3.4. Chemical Analysis

The legal quality parameters such as free acidity (FA), peroxide value (PV), UV spectrophotometric indices (K_232_, K_268_, ΔK), and fatty acid composition were evaluated by mean of the analytical procedures described in [36]. Analysis of tocopherols was performed following the method described in [37]. Analysis of hydrophilic phenolic compounds was performed following the analytical method described by [38].

### 3.5. Data Processing

All descriptive statistics as well as developing the NVS were performed using EXCEL in-house routines (Microsoft 365 version). Box-and-whiskers plots were drawn by mean of OriginPro 2018 (OriginLab Corporation, Northampton, MA USA http://www.originlab.com (accessed on 15 January 2024).

## 4. Conclusions

Due to the current lack, a scoring algorithm was proposed to rank the nutritional value of EVOOs based on their sensitivity to oxidation and composition in macronutrients and micronutrients, such as phenolic compounds and tocopherols. In order to ensure the universal validity of the a scoring algorithm, the variability of chemical data of the selected positive and negative components was evaluated from more than 1000 EVOO samples, from different producing countries (including the main producing countries worldwide, in addition to Australia) and from different production years to represent the highest possible number of production realities. On this basis, the algorithm for the nutritional value score (NVS) was defined, which only applies for samples of the EVOO category and allows the oils to be ranked on a scale of 0 (the worst possible nutritional value) to 100 (the best possible nutritional value).

Therefore, the scoring algorithm was designed to be an objective and useful tool to quantify the nutritional value of an EVOO as linked to an optimal balance of the chemical parameters that define the nutritional value itself. The choice of positive and negative chemical parameters was based on knowledge from the literature. Sensory properties were not directly involved in the algorithm, but were considered indirectly, since the minimum requirement for an oil to be evaluated is that it belongs to the EVOO category.

The use of the algorithm for calculation of the NVS might trigger producers and researchers to perform those chemical analysis that much of the scientific community to date neglects.

An additional benefit that the use of the NVS might induce is to refer to the EVOO category when the nutritional properties are the focus of the attention. In fact, the scientific literature too often refers to olive oil in a generic way, not taking into account that olive oils do not possess many of the nutritional properties typical of the EVOO.

## Figures and Tables

**Table 1:** Percentages (%) of the virgin olive oil samples within specific ranges of (**A**) free acidity, (**B**) peroxide value, (**C**) K_232_, (**D**) K_270_.

(A) Free Acidity (%)	2013 Samples	2014 Samples	Commercial Samples	(B) Peroxide Value (meq_O2_/kg)	2013 Samples	2014 Samples	Commercial Samples
0–0.15	4.8%	2.7%	8.9%	0–3.0	7.1%	2.9%	0.0%
0.15–0.25	74.1%	15.0%	44.5%	3.0–6.0	65.7%	30.9%	15.0%
0.25–0.4	19.4%	36.4%	34.9%	6.0–10.0	24.6%	45.7%	65.3%
0.4–0.5	0.8%	10.0%	8.9%	10.0–15.0	2.3%	17.1%	17.7%
0.5–0.6	0.3%	9.1%	2.1%	15.0–20.0	0.3%	2.9%	2.0%
0.6–0.8	0.0%	12.7%	0.7%	>20.0	0.0%	0.5%	0.0%
>0.8	0.6%	14.1%	0.0%				
**(C) K_232_**	**2013** **Samples**	**2014** **Samples**	**Commercial** **Samples**	**(D) K_270_**	**2013** **Samples**	**2014** **Samples**	**Commercial** **Samples**
0.00–1.50	5.5%	1.3%	0.0%	0.00–0.10	3.4%	3.9%	3.4%
1.50–1.65	20.9%	9.2%	3.4%	0.10–0.13	35.3%	17.1%	32.0%
1.65–1.85	56.1%	34.2%	24.5%	0.13–0.17	51.0%	51.2%	43.5%
1.85–2.15	16.6%	39.5%	49.0%	0.17–0.20	9.4%	23.7%	15.0%
2.15–2.50	0.9%	13.2%	17.7%	0.20–0.22	0.9%	1.3%	3.4%
>2.50	0.0%	2.6%	5.4%	>0.22	0.0%	2.6%	2.7%

**Table 2:** Percentages (%) of samples within specific ranges of (**A**) oleic acid, (**B**) linoleic acid, (**C**) saturated fatty acids (i.e., palmitic + stearic acids), (**D**) oleic/(linoleic + 2 × linolenic), for the samples from an olive oil production year free from adverse conditions (i.e., the 2013 samples), for the samples from an olive oil production year characterized by a strong olive fruit fly attack (i.e., the 2014 samples), and for the commercial samples, including oils from different geographical origins (i.e., Australia, Greece, Italy, Portugal, Spain, Tunisia).

(A). Oleic Acid (%)	2013 Samples	2014 Samples	Commercial Samples	Australia	Greece	Italy	Portugal	Spain	Tunisia
<55%	1%	0%	0%	0%	0%	0%	0%	0%	0%
55–63%	2%	2%	1%	0%	0%	0%	0%	1%	79%
63–71%	9%	3%	9%	31%	0%	3%	50%	11%	21%
71–78%	68%	53%	76%	50%	97%	56%	42%	46%	0%
77–85%	19%	42%	14%	19%	3%	41%	8%	42%	0%
> 85%	0%	0%	0%	0%	0%	0%	0%	0%	0%
**(B). Linoleic Acid (%)**	**2013** **Samples**	**2014** **Samples**	**Commercial** **Samples**	**Australia**	**Greece**	**Italy**	**Portugal**	**Spain**	**Tunisia**
<2.5%	0.0%	0.0%	0.0%	0.0%	0.0%	0.0%	0.0%	0.0%	0.0%
2.5–6%	34.1%	48.6%	18.3%	18.8%	8.3%	1.6%	9.7%	56.4%	0.0%
6–11%	58.4%	46.9%	78.0%	50.0%	91.7%	96.9%	66.1%	35.6%	0.0%
11–16%	4.6%	2.8%	3.7%	31.3%	0.0%	1.6%	24.2%	8.1%	64.3%
16–21%	2.9%	1.7%	0.0%	0.0%	0.0%	0.0%	0.0%	0.0%	35.7%
>21%	0.0%	0.0%	0.0%	0.0%	0.0%	0.0%	0.0%	0.0%	0.0%
**(C). Saturated Fatty Acids (%)**	**2013** **Samples**	**2014** **Samples**	**Commercial** **Samples**	**Australia**	**Greece**	**Italy**	**Portugal**	**Spain**	**Tunisia**
<7.5%	0.0%	0.0%	0.0%	0.0%	0.0%	0.0%	0.0%	0.0%	0.0%
7.5–11%	0.0%	0.0%	0.0%	0.0%	0.0%	0.0%	0.0%	0.0%	0.0%
11–15%	56.6%	81.0%	58.9%	62.5%	77.8%	90.5%	17.7%	63.5%	7.1%
15–20%	42.2%	18.4%	41.1%	37.5%	22.2%	9.5%	82.3%	36.5%	78.6%
20–25%	1.2%	0.6%	0.0%	0.0%	0.0%	0.0%	0.0%	0.0%	14.3%
>25%	0.0%	0.0%	0.0%	0.0%	0.0%	0.0%	0.0%	0.0%	0.0%
**(D). Oleic/(La + 2 × Lna)**	**2013** **Samples**	**2014** **Samples**	**Commercial** **Samples**	**Australia**	**Greece**	**Italy**	**Portugal**	**Spain**	**Tunisia**
<2.4	0%	1%	0%	0%	0%	0%	0%	0%	0%
2.4–4.5	3%	2%	3%	6%	0%	0%	0%	2%	100%
4.5–7.0	14%	5%	17%	31%	6%	6%	66%	14%	0%
7.0–14.0	82%	92%	76%	63%	94%	92%	27%	66%	0%
14.0–34.0	1%	0%	5%	0%	0%	2%	6%	18%	0%
>34.0	0%	0%	0%	0%	0%	0%	0%	0%	0%

**Table 3:** Percentages (%) of oils within specific ranges of (**A**) hydrophilic phenolic compounds and (**B**) tocopherols, for oil samples from an olive oil production year free from adverse conditions (i.e., the 2013 samples) and oil samples from an olive oil production year characterized by a strong olive fruit fly attack (i.e., the 2014 samples).

(A). Phenolic Compounds (mg/kg)	2013 Samples	2014 Samples
<50	0.2%	0.0%
50–150	5.0%	6.9%
150–250	15.6%	10.6%
250–350	23.9%	30.5%
350–500	31.9%	42.2%
500–650	18.4%	8.2%
650–800	4.3%	1.2%
800–1000	0.7%	0.2%
>1000	0.0%	0.2%
**(B). Tocopherols (mg/kg)**	**2013 Samples**	**2014 Samples**
<100	0.0%	2.8%
100–200	18.2%	54.2%
200–300	61.6%	31.7%
300–400	17.8%	11.1%
400–500	0.0%	0.0%
>500	2.4%	0.3%

**Table 4:** The mathematical model applied to the positive components of the NVS.

FATTY ACID COMPOSITION (%)—PC1	
Contribute to NVS score	$X_{\mathrm{PC}1}=X_{\mathrm{OA}}-X_{\mathrm{SFA}}-X_{\mathrm{Rat}}$
Contribute to X_PC1_	OA: $X_{\mathrm{OA}}=a\cdot {\left(\mathrm{OA}\right)}^{6}-b\cdot {\left(\mathrm{OA}\right)}^{5}+c\cdot {\left(\mathrm{OA}\right)}^{4}-d\cdot {\left(\mathrm{OA}\right)}^{3}+e\cdot {\left(\mathrm{OA}\right)}^{2}-f\cdot \left(\mathrm{OA}\right)+g$
	SFA: $X_{\mathrm{SFA}}=a\cdot {\left(\mathrm{OA}\right)}^{6}-b\cdot {\left(\mathrm{OA}\right)}^{5}-c\cdot {\left(\mathrm{OA}\right)}^{4}+d\cdot {\left(\mathrm{OA}\right)}^{3}-e\cdot {\left(\mathrm{OA}\right)}^{2}+f\cdot \left(\mathrm{OA}\right)-g$
	Rat: $X_{\mathrm{RAT}}=\;a\cdot {\left(\mathrm{Rat}\right)}^{6}-b\cdot {\left(\mathrm{Rat}\right)}^{5}+c\cdot {\left(\mathrm{Rat}\right)}^{4}-d\cdot {\left(\mathrm{Rat}\right)}^{3}+e\cdot {\left(\mathrm{Rat}\right)}^{2}-f\cdot \left(\mathrm{Rat}\right)+g$
PHENOLIC COMPOUNDS (mg/kg)—PC2	
Contribute to NVS score	If PC2 ≤ 250 mg/kg: $Y_{\mathrm{PC}2}=-a^{\prime }\cdot {\left(\mathrm{PC}2\right)}^{6}+b^{\prime }\cdot {\left(\mathrm{PC}2\right)}^{5}-c^{\prime }\cdot {\left(\mathrm{PC}2\right)}^{4}+d^{\prime }\cdot {\left(\mathrm{PC}2\right)}^{3}-e^{\prime }\cdot {\left(\mathrm{PC}2\right)}^{2}+f^{\prime }\cdot \;\left(\mathrm{PC}2\right)+g^{\prime }$
	If PC2 > 250 mg/kg: ${\;Y}_{\mathrm{PC}2}=-a^{\prime \prime }\cdot {\left(\mathrm{PC}2\right)}^{6}+b^{\prime \prime }\cdot {\left(\mathrm{PC}2\right)}^{5}-c^{\prime \prime }\cdot {\left(\mathrm{PC}2\right)}^{4}+d^{\prime \prime }\cdot {\left(\mathrm{PC}2\right)}^{3}-e^{\prime \prime }\cdot {\left(\mathrm{PC}2\right)}^{2}+f^{\prime \prime }\cdot \;\left(\mathrm{PC}2\right)+g^{\prime \prime }$
TOCOPHEROLS (mg/kg)—PC3	
Contribute to NVS score	$Z_{\mathrm{PC}3}=m\cdot \mathrm{PC}3+q$

X_PC1_, Y_PC2_, Z_PC3_: dimensionless values as functions of the content of the fatty acid composition (PC1), phenolic compounds (PC2), and tocopherols (PC3), respectively. PC1: fatty acid composition. PC2: phenolic compounds content in mg/kg. PC3: tocopherol content in mg/kg. X_OA_, X_SFA_, X_RAT_: dimensionless values as functions of the percentage content of oleic acid (OA), of the percentage content of saturated fatty acids (SFA), and of the value of the ratio OA/(LA + 2 × LnA) (Rat), respectively. OA: Oleic acid; SFA = (Palmitic + stearic acid): saturated fatty acids. Rat: = OA/(LA + 2 × LnA). a, b, c, d, e, f, g, a′, b′, c′, d′, e′, f′, g′, a″, b″, c″, d″, e″, f″, g″, m, q: coefficients of the equation developed.

**Table 5:** The mathematical model applied to the negative components of the *NVS*.

FREE ACIDITY (%)—NC1	
Contribute to NVS score	$X_{\mathrm{NC}1}=a\cdot \mathrm{NC}1^{6}-b\cdot \mathrm{NC}1^{5}+c\cdot \mathrm{NC}1^{4}-d\cdot \mathrm{NC}1^{3}+e\cdot \mathrm{NC}1^{2}-f\cdot \mathrm{NC}1+g$
PEROXIDE VALUE (meq_O2_/kg)—NC2	
Contribute to NVS score	$Y_{\mathrm{NC}2}=q+m\cdot \left(\mathrm{NC}2-k\right)$
UV SPECTROPHOTOMETRIC INDICES—NC3	
Contribute to NVS score	$Z_{\mathrm{NC}3}=Z_{K232}+Z_{K270}$
Contribute to Z_NC3_	$Z_{K232}=-a\cdot {\left(K_{232}\right)}^{6}+b\cdot {\left(K_{232}\right)}^{5}-c\cdot {\left(K_{232}\right)}^{4}+d\cdot {\left(K_{232}\right)}^{3}-e\cdot {\left(K_{232}\right)}^{2}+f\cdot \left(K_{232}\right)-g$
	$Z_{K270}=-a\cdot {\left(K_{270}\right)}^{6}+b\cdot {\left(K_{270}\right)}^{5}-c\cdot {\left(K_{270}\right)}^{4}+d\cdot {\left(K_{270}\right)}^{3}-e\cdot {\left(K_{270}\right)}^{2}+f\cdot \left(K_{270}\right)-g$

X_NC1_, Y_NC2_, Z_NC3_: dimensionless values as functions of the free acidity (NC1), peroxide value (NC2), and UV spectrophotometric indices (NC3), respectively. NC1: free acidity expressed as oleic acid (%). NC2: peroxide value expressed as meq_O2_/kg_oil_. NC3: UV spectrophotometric indices. Z_K232_: K_232_ value. Z_K270_: K_270_ value. a, b, c, d, e, f, g, k: coefficients of the equation developed.

## Data Availability

The data presented in this study are available in the article.
